# A randomized phase II trial of MR-guided prostate stereotactic body radiotherapy administered in 5 or 2 fractions for localized prostate cancer (FORT)

**DOI:** 10.1186/s12885-023-11430-z

**Published:** 2023-09-30

**Authors:** Sydney Wolfe, Marshall A. Diven, Ariel E. Marciscano, Xi Kathy Zhou, A. U. Kishan, M. L. Steinberg, Joseph A. Miccio, Philip Camilleri, Himanshu Nagar

**Affiliations:** 1 Department of Radiation Oncology, Weill Cornell Medicine/NewYork-Presbyterian, 525 East 68th Street, N-046, Box 169, New York, NY 10065 USA; 2 Department of Radiation Oncology, Brooklyn Methodist Hospital/New York-Presbyterian, Brooklyn, NY USA; 3 Division of Biostatistics, Department of Population Health Sciences, Weill Cornell Medicine/NewYork-Presbyterian, New York, NY USA; 4 Department of Radiation Oncology, University of California, Los Angeles, Los Angeles, CA USA; 5 Penn State Cancer Institute, Hershey, PA USA; 6 GenesisCare, Oxford, UK

**Keywords:** Prostate cancer (PCa), Definitive radiotherapy, Stereotactic body radiotherapy (SBRT), Magnetic resonance linear accelerator (MR-LINAC), MR-guided radiotherapy (MRgRT), Gastrointestinal (GI), Genitourinary (GU), Toxicity, Ultra-hypofractionation

## Abstract

**Background:**

Ultra-hypofractionated regimens for definitive prostate cancer (PCa) radiotherapy are increasingly utilized due in part to promising safety and efficacy data complemented by greater patient convenience from a treatment course requiring fewer sessions. As such, stereotactic body radiation therapy (SBRT) is rapidly emerging as a standard definitive treatment option for patients with localized PCa. The commercially available magnetic resonance linear accelerator (MR-LINAC) integrates MR imaging with radiation delivery, providing several theoretical advantages compared to computed tomography (CT)-guided radiotherapy. MR-LINAC technology facilitates improved visualization of the prostate, real-time intrafraction tracking of prostate and organs-at-risk (OAR), and online adaptive planning to account for target movement and anatomical changes. These features enable reduced treatment volume margins and improved sparing of surrounding OAR. The theoretical advantages of MR-guided radiotherapy (MRgRT) have recently been shown to significantly reduce rates of acute grade ≥ 2 GU toxicities as reported in the prospective randomized phase III MIRAGE trial, which compared MR-LINAC vs CT-based 5 fraction SBRT in patients with localized PCa (Kishan et al. JAMA Oncol 9:365-373, 2023). Thus, MR-LINAC SBRT–utilizing potentially fewer treatments–is warranted and clinically relevant for men with low or intermediate risk PCa electing for radiotherapy as definitive treatment.

**Methods/Design:**

A total of 136 men with treatment naïve low or intermediate risk PCa will be randomized in a 1:1 ratio to 5 or 2 fractions of MR-guided SBRT using permuted block randomization. Randomization is stratified by baseline Expanded PCa Index Composite (EPIC) bowel and urinary domain scores. Patients undergoing 5 fractions will receive 37.5 Gy to the prostate over 10–14 days and patients undergoing 2 fractions will receive 25 Gy to the prostate over 7–10 days. The co-primary endpoints are GI and GU toxicities as measured by change scores in the bowel and urinary EPIC domains, respectively. The change scores will be calculated as pre-treatment (baseline) score subtracted from the 2-year score.

**Discussion:**

FORT is an international, multi-institutional prospective randomized phase II trial evaluating whether MR-guided SBRT delivered in 2 fractions versus 5 fractions is non-inferior from a gastrointestinal (GI) and genitourinary (GU) toxicity standpoint at 2 years post-treatment in men with low or intermediate risk PCa.

**Trial registration:**

Clinicaltrials.gov identifier: NCT04984343. Date of registration: July 30, 2021.

Protocol version: 4.0, Nov 8, 2022.

**Supplementary Information:**

The online version contains supplementary material available at 10.1186/s12885-023-11430-z.

## Background

Prostate cancer (PCa) is the most diagnosed non-skin cancer among men and is the second leading cause of death from cancer in men behind lung/bronchus cancer [1]. For patients with localized PCa, standard guideline-based treatment options, such as those published by National Comprehensive Cancer Network (NCCN), include active surveillance, radical prostatectomy or definitive radiotherapy utilizing external beam radiation therapy (EBRT) and/or brachytherapy with or without androgen deprivation therapy (ADT) intensification. Specific recommendations are based on patients’ initial risk stratification, which factors in life expectancy, prostate specific antigen (PSA) levels, digital rectal exam results as well as pathology characteristics from systematic prostate biopsy (i.e., Gleason Score and extent of gland involvement). PCa is relatively unique among other malignancies due to favorable outcomes in men regardless of their choice for initial management. For instance, the recently published 15-year outcomes from the United Kingdom ProtecT trial, which randomized men diagnosed with localized PCa after screening PSA to either active monitoring, prostatectomy or radiotherapy, suggest low PCa-specific mortality (PCSM) regardless of the treatment assigned to patients [2]. Men who elect for or are recommended active treatment can decide between which primary modality of treatment to pursue. The decision is typically dependent upon patient preference after informed discussion regarding risk, benefits and expected acute and long-term side effects of the discussed local treatment options. In the case of definitive PCa treatment with prostatectomy or radiotherapy, the most affected domains are genitourinary (GU), gastrointestinal (GI) and sexual function with similar yet nuanced characteristics and expected risks specific to each approach. As such, evaluation of new radiation treatment regimens should focus on how the treatment affects these specific domains both acutely and chronically.

Stereotactic body radiation therapy (SBRT) is an ultra-hypofractionated EBRT treatment delivering between 36.25-45 Gy over 5 fractions, and it is increasingly used for certain PCa patients [3]. To date, prostate SBRT has shown excellent local control and low toxicity with a side effect profile on par with moderately hypofractionated (~ 4–5 weeks) and conventionally fractionated (~ 8–9 weeks) regimens. Several randomized prospective studies have confirmed that SBRT is a safe and reasonable patient-centric alternative to more protracted radiation schedules. The added convenience of fewer visits by utilizing shorter radiation regimens allows for greater access to treatment for patients and helps reduce financial toxicity and treatment burden on healthcare systems [4–6]. HYPO-RT-PC, a randomized phase III trial, compared ultra-hypofractionated (42.7 Gy over 7 fractions) to conventionally fractionated (78 Gy over 39 fractions) radiation treatment regimens and demonstrated non-inferior failure-free survival (FFS) of 84% in both treatment groups at 5-years. A wide range of radiotherapy techniques were permitted, including image-guided three-dimensional radiotherapy, intensity-modulated radiotherapy (IMRT), or volumetric modulated arc therapy (VMAT) planning and use of fiducial markers at discretion of treating physician [7]. Long-term urinary and bowel patient-reported quality of life (QOL) and physician-reported Radiation Therapy Oncology Group (RTOG) outcomes were similar between treatment arms despite significantly higher urinary and bowel patient-reported acute toxic outcomes [8]. The treatment planning employed in HYPO-RT-PC is largely outdated as technology has advanced, leading to smaller planning target volume (PTV) and adoption of IMRT/VMAT techniques over that of 3-dimensional conformal radiation therapy.

PACE-B, a randomized phase III trial, compared conventionally fractionated (78 Gy in 39 fractions) or moderately hypofractionated (62 Gy in 20 fractions) IMRT-based regimens to SBRT (36.25 Gy in 5 fractions) for low and favorable intermediate-risk PCa patients. Results from the study showed no significant differences in patient-reported outcomes for acute toxicities. Only physician-reported Common Terminology Criteria for Adverse Events (CTCAE) grade ≥ 2 severe GI toxicity was significantly worse in the SBRT group, however this difference resolved by week 12 following radiotherapy [9]. At 2 years post-treatment, RTOG GI and GU toxicity rates for SBRT and conventional schedules of radiotherapy were similar [10]. Use of fiducials for patients undergoing SBRT was not universal and the majority of patients undergoing SBRT did not have motion monitoring during treatment. Thus, more favorable toxicity profiles are attainable with more modern SBRT techniques. In addition to this prospective data, a large cohort study involving 2142 patients across 10 institutional sites and 2 multi-institutional trials reported that SBRT for low and intermediate-risk disease was associated with high rates of biochemical control and low rates of severe toxic events, with a 7-year cumulative incidence of severe grade ≥ 3 GU and GI toxic events of 2.4% and 0.4%, respectively [11]. Furthermore, rates of acute toxicities were relatively low with grade ≥ 2 GU and GI reported to be 9.6% and 3.4%, respectively. These results likely reflect the expected side effect profile of current SBRT standards not using MRI guidance, as the trials included in this consortium utilized fiducials in all patients with most treatment plans incorporating intrafraction motion monitoring or interval imaging to account for anatomic changes during course of treatment.

In addition to the safe side effect profile, practical advantages, and patient-centric convenience of ultra-hypofractionated radiation treatment regimens, there is also biological rationale to provide definitive treatment in 5 or potentially fewer fractions. Among solid tumors, PCa appears unique in that ultra-hypofractionated SBRT-like regimens are hypothesized to expand the therapeutic window between tumor control and late toxicity (due to a purported low α/β ratio) [12, 13]. Thus, it is important to explore whether further hypofractionation is possible without compromising tumor control or increasing acute and/or long-term treatment related toxicities. In support of the notion that ultra-hypofractionation is therapeutically advantageous for PCa, high dose-rate (HDR) brachytherapy is commonly delivered over one to four fractions, either as definitive treatment or in conjunction with EBRT, and has an excellent track record of local control. In a randomized phase II trial comparing one fraction of 19 Gy to 2 fractions of 13.5 Gy using HDR brachytherapy for low and intermediate-risk PCa patients, the 5-year biochemical disease-free survival and cumulative incidence of local failure (LF) was 73.5% and 29% in the single fraction arm and 95% (*p* = 0.001) and 3% (*p* < 0.001) in the 2-fraction arm, respectively. Grade 2 late rectal toxicity occurred in 1% while the incidence of grade 2 and 3 urinary toxicity was 45% and 1%, respectively, with no difference between arms. While single fraction monotherapy was inferior and should not be used, these data demonstrate that brachytherapy can be safely delivered as 2 high dose fractions with a high cancer control rate at 5 years [14]. A single arm prospective clinical trial (2STAR) from Sunnybrook reporting on 30 patients with low (10%), favorable intermediate (33%) and unfavorable intermediate-risk (57%) cancer demonstrated the safety and feasibility of a 2-fraction approach to computed tomography (CT)-based SBRT for this patient demographic. Of these 30 patients, 6 (20.7%) had a minimal clinically important change (MCIC) in the urinary domain, 6 (21.4%) had a MCIC in the bowel domain, and 3 (20%) had a MCIC in the sexual domain at follow-up (median 49.5 months) [15]. Taken together, these data provide rationale for investigating the potential to deliver definitive SBRT treatment in fewer fractions without increasing side effects while maintaining adequate therapeutic effectiveness.

With technological advances that improve the visualization of targets and precision of radiation delivery during treatment, higher doses can be delivered in fewer fractions with less side effects. The integration of magnetic resonance (MR)-imaging with linear accelerator (LINAC) technology — available with MR-LINAC — allows for better visualization of the prostate and reduces planning treatment volumes, while also enabling real-time tracking of targets and organs-at-risk (OAR) throughout the course of radiation delivery [14, 16]. Taken together, these features theoretically allow for treatment plans that are advantageously adaptive and responsive to individualized anatomical changes during treatment and throughout the treatment course enabling greater precision and OAR sparing.

The theoretical of advantages of MR-guided radiotherapy (MRgRT) are being evaluated on clinical trials including the phase III MIRAGE trial which is comparing standard CT-guided SBRT and MR-guided SBRT for PCa. Interim results from the trial showed that acute grade ≥ 2 GU and GI toxicities were significantly reduced in men receiving MR-guided treatment (24.4% vs. 43.4%, *p* = 0.01 and 0% vs. 10%, *p* = 0.003, respectively). In terms of patient reported outcomes, the group receiving MR-guided SBRT had fewer patients with 15-point or higher increase in International Prostatism Symptom Score (IPSS) at one month (6.8% vs. 19.4%, *p* = 0.01) and fewer patients that reported a clinically significant reduction in Expanded Prostate Cancer Index Composite (EPIC) bowel (25% vs. 50%, *p* = 0.001) and urinary incontinence scores [17, 18]. These interim results suggest that the uncertainty margin reduction enabled by MR-LINAC based technology has clear clinical value and can enhance patient outcomes by reducing acute treatment related toxicities compared to that of the current CT-guided SBRT. This trial may well set the standard benchmark for treatment related toxicities for men undergoing SBRT for localized PCa. Of note, the HERMES study (NCT04595019) has started enrolling subjects to compare 2 versus 5 fractions of MR-guided SBRT using a similar dose-fractionation schedule to that which our FORT trial employs (https://www.clinicaltrials.gov/ct2/show/NCT04595019).

Thus, the FORT trial (NCT04984343) is designed to evaluate whether MR-LINAC SBRT delivered in 2 fractions has a non-inferior GU and GI toxicity profile as compared with MR-LINAC SBRT delivered in 5 fractions among patients with low to intermediate-risk PCa.

## Methods/Design

This is a phase II non-blinded multi-institution randomized non-inferiority trial comparing patient reported outcomes in GI or GU toxicity at baseline and 24 months post treatment in men with low or intermediate risk PCa receiving either 2 or 5 fraction MR-LINAC guided SBRT. Acute and late patient reported changes in GI, GU and sexual symptoms as well as time to progression (TTP) and overall survival (OS) will be evaluated at 3, 6, 12, 24 and 60 months post-treatment. We plan to enroll 136 patients with an expected rate of accrual of approximately 120 patients per year. MR-LINAC SBRT will be delivered with 37.5 Gy in 5 fractions or 25 Gy in 2 fractions to the prostate ± seminal vesicles, with an optional simultaneous integrated boost (SIB) of up to 45 Gy for the 5 fraction arm and up to 28 Gy for the 2 fraction arm. Patients will be randomized to treatment arms in a 1:1 ratio using a computer-generated randomization scheme. Patients on the 5 fractions arm will receive their radiation on non-consecutive days. Patients on the 2 fraction arm will be treated with at least 6 days between treatments. For both arms, treatment should ideally be completed by 2 weeks post-start date.

### Objectives

The primary objective is to demonstrate that 2 treatments of radiotherapy does not significantly increase patient-reported GI and GU symptoms compared to 5 treatments of radiotherapy 2 years after treatment completion.

#### Primary endpoint

The primary endpoint is the change in the number of patient-reported GI and GU using the EPIC at baseline and 24 months from the end of radiation treatment.

#### Secondary endpoints

1. Compare patient reported GI symptoms using the EPIC at the end of treatment and 3, 6, 12, 24 and 60 months from the end of treatment.
2. Compare patient reported GU symptoms using the EPIC at the end of treatment and 3, 6, 12, 24 and 60 months from the end of treatment.
3. Compare TTP where progression is defined as the first occurrence of biochemical failure (BF), LF, regional failure (RF), distant metastasis (DM), institution of new unplanned anticancer treatment, or PCSM.
4. Compare freedom from biochemical failure (FFBF) and TTP rates with an alternate PSA ≥ PSA nadir + 2 ng/mL definition of BF.
5. Compare LF, RF, salvage therapy (i.e. institution of new unplanned anticancer treatment), DM, PCSM and OS rates.

#### Inclusion criteria

- Men aged ≥ 18 with histologically confirmed low or intermediate risk PCa per NCCN guidelines
- Eastern Cooperative Oncology Group (ECOG) score of 0 – 1
- IPSS < 18
- Ability to receive MR-guided radiotherapy
- Ability to complete the EPIC questionnaire
- Patients with a prior or concurrent disease whose natural history or treatment does not have the potential to interfere with the safety or efficacy assessment of the investigational regimen are eligible for this trial. Note: Any patient with a cancer (other than keratinocyte carcinoma or carcinoma in situ or low-grade non-muscle invasive bladder cancer) who has been disease-free for less than 3 years must contact the Principal Investigator.

#### Exclusion criteria

- Prior history of receiving pelvic radiotherapy
- Patient with history of inflammatory bowel disease
- MRI Prostate Volume > 80 cc
- MRI Stage > T3a
- Unilateral or bilateral hip replacements
- History of bladder neck or urethral stricture
- Transurethral resection of the prostate (TURP) < 8 weeks prior to radiotherapy
- Metastatic (pelvic nodal or distant) disease on CT, Bone, Fluciclovine, and/or prostate-specific membrane antigen (PSMA) positron emission tomography (PET) scan

### Patient selection, study enrollment, and randomization and blinding

Patients being evaluated for potential treatment of their PCa with definitive radiotherapy will be informed of their eligibility to participate in this clinical trial. An informed consent form will be provided to all patients considering participation in this clinical for personal review. Patients who agree to participate in the study will sign the informed consent form and be provided with a copy of the signed document. Participants will also have the option to be consented remotely using an electronic version of the informed consent form through the Research Electronic Data Capture (REDCap) platform in accordance with federal, state and local regulations, as applicable.

**Fig. 1:**
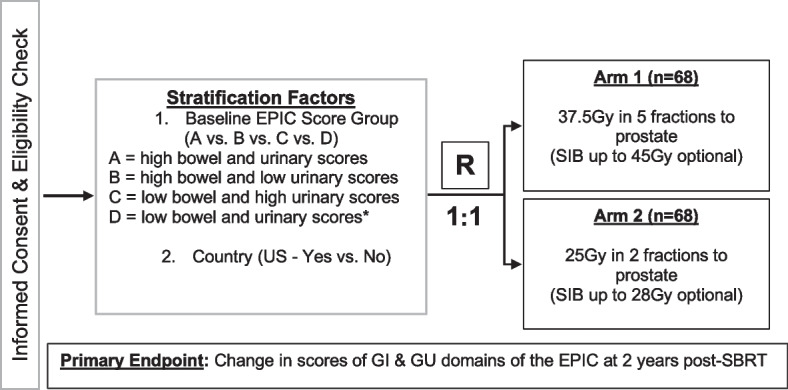
Trial Schema. Expanded PCa Index Composite (EPIC), EuroQol-5D Index (EQ-5D) and International Prostate Symptom Score (IPSS) quality of life (QOL) surveys are collected at screening, baseline (before or first day of radiotherapy), end of radiotherapy, and at 3 month, 6 month, 9 month, 12 month, 15 month, 18 month, 21 month, 24 month and 60 month follow-up visits. *EPIC score groups determined as: high bowel score > 96, low bowel score ≤ 96, high urinary score > 84, low urinary score ≤ 84. ** GI, gastrointestinal; GU, genitourinary; RT, radiotherapy

This study will employ a phase II non-inferiority design to compare 5 fractions of ultra-hypofractionated radiotherapy versus 2 fractions of ultra-hypofractionated radiotherapy in the definitive setting for PCa. Subjects will be stratified according to baseline EPIC bowel and urinary domain scores (high bowel score and high urinary score vs. high bowel score and low urinary score vs. low bowel score and high urinary score vs. low bowel score and low urinary score) and treatment country (US vs. other). Participants in each stratum will be randomized to the 25 Gy in 2 fraction or 37.5 Gy in 5 fraction treatment arms in a 1:1 ratio using permuted block randomization with varying block sizes as outlined in trial schema shown in Fig. 1.

Patients and study investigators will not be blinded given the types of treatments being evaluated and data management will be performed in online clinical trial database. All trial participants, physicians/ health care providers, data analyzers and symptom assessors will be aware of treatment assignment. Randomization will be communicated by email or phone to treating physician and conveyed to patients in a similar manner.

### Interventions

#### Radiation treatment planning

After consent, eligibility verification and randomization, patients will undergo CT/MRI simulation and radiotherapy planning for treatment on MR-LINAC. Patients may receive placement of rectal spacer prior to CT/MRI planning scan at the discretion of the treating physician. Patients will receive treatment to the prostate ± seminal vesicles as per the treating physician’s discretion, either with 37.5 Gy in 5 fractions or 25 Gy in 2 fractions (see Table 1 for radiotherapy prescription dose rational based on biologically effective dose (BED) and equivalence dose (EQD2) comparison). An optional SIB may be administered as per the treating physician’s discretion, with doses up to 45 Gy for the 5 fraction arm and up to 28 Gy for the 2 fraction arm. The SIB will be concordant with PSMA and MRI imaging and biopsy findings. There will be no PTV expansion for the boost volume. Subjects on the 5 fraction arm should be treated on non-consecutive days. Subjects on the 2 fraction arm must have at least 6 days between treatments and it is suggested that patients receive treatment on the same day of subsequent weeks (e.g. treatment on Monday and the following Monday). The goal is for treatment to be completed within 2 weeks of starting for both arms.

**Table 1:** Biologically Effective Dose (BED) and Equivalence Dose (EQD2) comparison between 5 and 2 fraction schedules

Comparison of 7.5 Gy × 5 (37.5 Gy) to 12.5 Gy × 2 (25 Gy)						
**Organ**	**Dose (Gy)**	**BED (Gy)**	**EQD2 (Gy)**	**Dose (Gy)**	**BED (Gy)**	**EQD2 (Gy)**
Prostate (α/β = 2.7)	37.5 (7.5 Gy × 5)	141	81	25 (12.5 Gy × 2)	141	81
Bladder (α/β = 3)	37.5 (7.5 Gy × 5)	131	79	25 (12.5 Gy × 2)	128	79
Rectum (α/β = 5)	37.5 (7.5 Gy × 5)	94	67	25 (12.5 Gy × 2)	88	63

##### Contours

1. The prostate ± seminal vesicles will be contoured as the clinical target volume (CTV). The PTV expansion for the CTV will be 2–3 mm at the physician’s discretion.
2. The optional boost volume will be at the discretion of the treating physician, but the addition of a boost should not exceed dose constraints compared to a non-boost plan.
3. The rectum will be drawn from the bottom of the ischial tuberosities to the sigmoid flexure.
4. The bladder, bladder wall (bladder—4 mm isotropic constriction), urethra (method of urethral delineation is at the treating physician’s discretion), femoral heads, and penile bulb will also be contoured as normal structures. Delineation of the urethra can be performed using the T2 sequence, contouring and interpolating on axial slices where the urethra is visible, and/or contouring on the sagittal plane from the bladder to the penile urethra.

##### Treatment dose planning parameters for 37.5 Gy in 5 fractions

The PTV will be treated with the prescribed dose of 37.5 Gy in 5 fractions. The volume of the PTV receiving the Prescription Dose (VPrescription Dose) of 37.5 Gy should be ≥ 95% and not exceed 125% (hotspot). The urethra should be expanded with a 3 mm planning organs-at-risk volume (PRV) and should not receive more than a point dose of 110% of the prescription dose of 37.5 Gy (see Additional file 1).

##### Treatment dose planning parameters for 25 Gy in 2 fractions

The PTV will be treated with the prescribed dose of 25 Gy in 2 fractions. The volume of the PTV receiving the Prescription Dose (VPrescription Dose) of 25 Gy should be ≥ 95% and not exceed 125% (hotspot). The urethra should be expanded with a 3 mm PRV and should not receive more than a point dose of 110% of the prescription dose of 25 Gy (see Additional file 1).

##### Adaptive planning

1. Adaptive planning is mandatory for all subjects on the 2 fraction arm. The need for adaptive planning will be assessed for all subjects on the 5 fraction arm on a per treatment basis.
2. Prior to treatment, each patient will undergo a scout/set-up MRI scan followed by the treatment planning quality (~ 3 min) true fast imaging with steady-state free progression (TRUFI) scan.
3. 2D shifts will be performed to align relevant anatomy (bladder, rectum, prostate ± seminal vesicles).
4. Simulation contours will be rigidly copied to the set-up scan and recontoured if CTV or boost (optional) delineation changes. OAR should be contoured within a 3 cm contouring ring.
5. Predict dose algorithm will determine if treatment dose parameters meet planning dose parameters.
6. Patients will undergo adaptive planning if treatment dose parameters do not meet planning parameters and protocol mandatory planning parameters (see Additional file 1).

##### Treatment delivery and gating

1. A short scan (approximately 25 s) should be used for set-up and assessment of appropriate bladder fill and rectal position.
2. A long scan (approximately 3 min) should be used for shifts prior to treatment delivery.
3. A 2 mm tracking boundary should be used with 5% region of interest for gating during treatment delivery.

##### Concomitant medication and supportive care guidelines

1. ADT for 4–6 months duration may be prescribed at the investigator’s discretion and/or patient’s choice. ADT can be administered in a neoadjuvant (up to 2 months prior to radiotherapy), concurrent, or adjuvant (after the last fraction of radiotherapy) manner.
2. A proportion of patients undergoing prostate radiotherapy can expect an increase in urinary frequency or urgency. If this becomes bothersome to the patient, medication to alleviate symptoms can be prescribed at the discretion of the treating radiation oncologist and documented in the patient chart.
3. Serious bowel symptoms during the time of prostate radiotherapy are rare. If patients develop rectal urgency, tenesmus or diarrhea, medication to alleviate symptoms can be prescribed at the discretion of the treating radiation oncologist and documented in the patient chart.

#### Trial procedures

The trial procedures to be performed at each patient visit as per FORT protocol are outlined in Table 2. (Note: post-radiation follow-ups have a ± 1 month window).

**Table 2:** Schedule of trial events

Procedure	Screening	First Day of RT	Last Day of RT	FUP at 3, 9, 15, 18, 21 mo post-RT	FUP at 6, 60 mo post-RT	FUP at 12 mo post-RT	FUP at 24 mo post-RT
Informed Consent	X						
Demographics/MH	X						
Physical Exam	X			X	X	X	X
VS, Height, Weight	X			X	X	X	X
PSA	X			X	X	X	X
Pelvic MRI	X					X (Optional)	X (Optional)
PSMA PET Scan^a^	X					X	X
Decipher GC Score	X						
Whole Blood, Serum, Plasma (Optional)		X	X		X	X	X
Urodynamic Testing (Optional)		X	X				X
EPIC	X	X	X	X	X	X	X
IPSS	X	X	X	X	X	X	X
EQ-5D	X	X	X	X	X	X	X
CTCAE v5.0	X	X	X	X	X	X	X
Post-Treatment Biopsy (Optional)							X

^a^Screening PSMA PET is according to indication of use in men with PCa suspected metastasis who are candidates for initial definitive therapy. PSMA PETs at follow-up are with piflufolastat F 18

*RT* salvage radiotherapy, *FUP* follow up visit, *mo* months, *MH* medical history, *VS* vital signs, *PSA* prostate-specific antigen, *MRI* magnetic resonance imaging, *PSMA PET* prostate-specific membrane antigen positron emission tomography, *Decipher GC Score* Decipher Genomic Classifier Score, *EPIC* Expanded PCa Index Composite, *IPSS* International Prostate Symptom Score, *EQ-5D* EuroQol-5D Index, *CTCAE* Common Terminology Criteria for Adverse Events version 5.0

### Follow-up phase

After completing radiation therapy as per the study protocol, patients will be seen for follow-ups every 3 months for the first 2 years following treatment and at 5 years post-treatment. At each of these time points, patients will be asked to complete the EPIC, IPSS and EuroQol-5D Index (EQ-5D) QOL questionnaires.

The EPIC questionnaire is used to evaluate 5 symptom domains relating to PCa treatment including urinary incontinence, irritative or obstructive urinary symptoms, sexual symptoms, bowel symptoms and hormonal symptoms with scoring on a scale from 0 (worst) to 100 (best) [19].

The IPSS questionnaire is used to evaluate lower urinary tract symptom severity with scores 0–7 representing mild symptoms, scores 8–19 representing moderate symptoms, and scores 20–35 representing severe symptoms. The IPSS also includes one question to reflect the patient’s overall urinary quality of life with answers ranging from 0 (“delighted”) to 6 (“terrible”) [20].

The EQ-5D questionnaire is a validated instrument with QOL assessments across 5 dimensions including mobility, self-care, usual activities, pain/discomfort, and anxiety/depression. Each dimension is graded from levels 1–5, with level 1 indicating no problems, level 2 indicating slight problems, level 3 indicating moderate problems, level 4 indicating severe problems and level 5 indicating an inability to perform/extreme problems. The EQ-5D also includes a visual analog scale that assesses overall health. The scores across the 5 dimensions are transformed into an index utility score between 0 (“worst health state”) and 1 (“best health state”) [21]. This study will report the multidimensional utilities for comparative purposes. The measured utility values for each patient on this study will be combined with overall survival to calculate the quality-adjusted life years. This will then be used for cost-effectiveness analysis comparing the two arms in this trial.

### Adverse events

The descriptions and grading scales found in the CTCAE v5.0 will be utilized for reporting of all adverse events (AEs). All AEs and serious adverse events (SAEs) that occur on this study will be reported to the Institutional Review Board (IRB) of Weill Cornell Medicine according to IRB policy. All AEs and SAEs reported during this study will be followed until resolution or stabilization.

### Data management and safety monitoring

REDCap will be used to collect and maintain all treatment, toxicity, efficacy, and AE data for all enrolled subjects. REDCap is a secure data management software system that is fully supported by Weill Cornell Medicine’s Clinical and Translational Science Center (CTSC). REDCap provides audit trails that track the creation and modification of records that include user identification and timestamp. Once data is entered into the system, the data is subject to validation procedures that are executed immediately, upon saving the electronic case report form page, or during the batch validation process. Validation failures will generate a discrepancy that can be corrected by data managers or the project data manager depending on the database account privileges. Once the discrepancy is corrected, the data is marked as clean, and an audit trail is recorded by the system.

Security measures that will be taken to protect patient data will include firewall technology and database level security which will be achieved by assigning roles and privileges to different levels of users and by requiring that the users authenticate themselves using user identification and password. Additional security for data transfer between remote clients and servers will be achieved by using digital certificates. All data will be backed-up to tape periodically according to the institutional standard operating procedures. All data will be stored for at least 5 years following the termination of the study.

The Weill Cornell Medicine Data Safety Monitoring Board (DSMB) will serve as the central monitoring board for the study. An independent medical monitor from the Weill Cornell Medicine DSMB will review cumulative study data twice a year to evaluate safety, efficacy, study conduct, and scientific validity and integrity of the study. This study will be conducted in accordance with the guidelines in the 2001 National Cancer Institute-approved data and safety monitoring plan for the Weill Cornell Medicine Meyer Cancer Center. Reports to the DSMB will include information regarding accruals, targets, responses, AEs, and evidence of reporting to appropriate review committees. Safety reports will be submitted to the DSMB every 6 months for review. The Weill Cornell Medicine DSMB will review the IRB-approved protocol, the data and safety monitoring plan and any stopping guidelines during protocol initiation. The study protocol is approved by the IRB of Weill Cornell Medicine and all methods in this study are conducted in accordance with IRB guidelines, rules, and regulations.

### Statistical analysis

#### Sample size and accrual

The primary goal of this study is to determine if 2 fractions of MRI-LINAC SBRT does not increase GI or GU toxicity over 5 fractions of radiotherapy at 2 years post treatment. The primary endpoints are change scores in the GI and GU domains per EPIC questionnaire. The change scores will be based on the 2-year score minus the pretreatment (baseline or before radiation therapy) score. The hypothesis for this endpoint is that the EPIC mean change score is no worse in the 2 fraction arm than it is in the 5 fraction arm for either type of toxicity.

The sample size is calculated based on a non-inferiority design. The non-inferiority margins are set to be a change score of 6 points for the GI symptoms and 5 points for the GU symptoms. The standard deviations of the change scores are assumed to be 13.2 for the GI symptoms and 10.5 for the GU symptoms based on estimates generated in the RTOG 0415 trial [22]. This level of change in scores is deemed as clinically meaningful. For example, 6 points of change score for GI symptoms corresponds to 2 symptoms worsening by 1 level (i.e. loose stools and frequency of bowel movements change from “no problem” to “very small problem”) or 1 of the symptoms worsening by 2 levels (i.e. loose stool change from “no problem” to “small problem”).

A sample size of 122 with 61 in each arm will ensure 80% power for GI endpoint and 83% power for GU endpoint to detect non-inferiority using a one-sided two-sample t-test at the significance level of 0.05. Adjusting for a projected 10% EPIC/non-compliance rate, 136 patients (68 per arm) will be randomized.

The projected accrual rate is 10 patients per month. Based on this information, it is projected that the study will complete accrual in about 18 months. The primary endpoint analysis will occur approximately 4 years from study activation.

#### Data analysis

##### Analysis of primary endpoints

The co-primary endpoints are GI and GU toxicity as measured by the bowel and urinary EPIC domains, respectively. The change scores, calculated as baseline score subtracted from 2-year score, will be analyzed using a non-inferiority t-test based on the prespecified non-inferiority margins with a significance level of 0.05. If the data are determined to be non-normal, a Wilcoxon test may be used instead. All patients with EPIC bowel and urinary domain scores will be included in the primary endpoint analysis. The EPIC scoring manual will be followed which requires ≥ 80% of items in a domain to be completed to obtain a score for that domain. Stratification variables (baseline EPIC score and country of treatment), ADT use, rectal spacer use, SIB use, age, race, and other covariates (Gleason, T-stage), will be adjusted for as appropriate in this analysis. This is the same primary endpoint analysis as used in the SHORTER trial [23].

##### Early stopping guidelines/interim futility analysis

An interim futility analysis will be carried out when 1/3 of patients have 6 months follow-up. If the upper 95% confidence limit of the mean difference in 6-months change scores between the treatment arms is less than the pre-specified non-inferiority margins, i.e., < -6 for GU and < -5 for GI, the 2 fraction arm will be deemed inferior to the 5 fraction arm. This is the same interim futility analysis as used in the SHORTER trial [23]. Early reporting of the treatment results will be recommended to the DSMB, who will review these results. This approach has a minimal effect on statistical power.

##### Analysis of secondary endpoints

- *Secondary safety endpoints:* All safety endpoints as measured by change in EPIC scores will be calculated as baseline score subtracted from follow-up score. Differences between study arms will be examined using t-test or Wilcoxon rank sum test where appropriate. The follow-up time points of interest are the end of radiotherapy, 3, 6, 12, 24 and 60 months from the start of treatment. A longitudinal analysis incorporating all follow-up time points, will be conducted separately for each domain score using a linear mixed-effects model, adjusting for baseline domain score, treatment arm, Gleason score, baseline PSA, T-stage, age, and race. These analyses will be conducted regardless of the outcomes of the primary t-test. For the comparison of primary endpoints at 2 years adjusting for other variables using, especially stratification variables, the analysis of covariance (ANOVA) will be used. The results from the covariate adjusted analysis are expected to be similar to those of the primary analyses. In the rare case of different results, the impact of missing data and covariates will be examined to help make meaningful conclusions. If any of the domains are found to differ significantly between arms, analysis of that domain’s subscales will be undertaken to assess which particular subscale is driving the significant difference. The subscales function in terms of both incontinency and irritative/obstructive for the urinary domain, and function and bother for the bowel domain. The impact of the missing data on the analysis results will be evaluated using multiple strategies including: 1) adjustment of patient characteristics that are associated with missingness at any time point for the EPIC bowel and urinary domain scores in the mixed effects model assuming that the data are missing at random (MAR); 2) using a joint model that allows a shared parameter between the repeated measurements and time to death or drop out if data are found to be missing not at random (MNAR) due to the high number of patient deaths or dropouts or using pattern mixture and selection models [24–27]. Sensitivity analyses will be performed to compare the results of different analytic strategies [28]. This is the same secondary safety endpoint analysis as used in the SHORTER trial [23].
- *Secondary efficacy endpoints:* For competing-risk endpoints such as PCSM, LF, RF, TTP, and DM, Gray’s cumulative incidence method will be used with death as a competing risk for LF, RF and DM and death not due to PCa for PCSM and TTP. OS and FFBF will be estimated by the Kaplan–Meier method and compared between arms with the log-rank test. Cox regression will be used to obtain hazard ratios (HRs) for OS and TTP. Fine and Gray’s regression will be used for the endpoints with competing risks. Adjusted HRs and the respective 95% confidence interval will be computed. Baseline PSA, stratification variables (baseline EPIC score and country of treatment), ADT use, rectal spacer use, SIB use, age, race, and other covariates (Gleason, T-stage) will be adjusted for as appropriate in this analysis. This is the same secondary efficacy endpoint analysis as used in the SHORTER trial [23].

## Discussion

Historically, radiotherapy for PCa has consisted of smaller doses of radiation being delivered over longer courses of treatment to minimize toxicity to adjacent internal organs, such as the bladder, bowel and rectum, while delivering a biologically effective dose to treat malignant disease. With innovations in radiation treatment planning and delivery along with evidence supporting low α/β ratio of PCa, it has become possible to deliver higher doses of radiation in fewer fractions without sacrificing patient safety and clinical efficacy. This shift toward hypofractionation and even ultra-hypofractionation with SBRT is of great benefit to patients and health care systems, as it enables them to make fewer trips to their treatment center sparing added financial toxicity and resource utilization associated with more protracted courses. The advent of MR-based planning and radiation delivery is one such technological advancement that allows for hypofractionated and ultra-hypofractionated radiotherapy regimens with favorable OAR sparing. Specifically, MR-guided radiotherapy enhances visualization of and localization to the target volumes while also allowing online adaptive planning and monitoring of the patient's physiologic motion in real-time. Taken together, ultra-hypofractionation and MR-guidance have the capacity to greatly improve patients’ quality of life both during and after their treatment. The FORT trial is one of few trials currently evaluating whether MR-guidance can effectively minimize toxicity amidst an ultra-hypofractionated radiation treatment regimen for definitive management of localized PCa. We hope that the data from this trial elucidates the non-inferiority of 2 vs. 5 ultra-hypofractionated MR-guided radiotherapy allowing for new therapeutic options for men with low or intermediate risk PCa.

### Supplementary Information

**Additional file 1: Supplemental Table 1.** MR-guided Prostate Stereotactic Body Radiation Therapy Organ-at-Risk Constraints and Planning Parameters for 2-fraction and 5-fraction Regimens.

## Data Availability

The datasets generated and/or analyzed during the current study are not publicly available due to the ongoing nature of the trial and possible compromise of individual privacy but are available from the corresponding author upon reasonable request.

## Abbreviations

PCa: Prostate cancer
SBRT: Stereotactic body radiation therapy
FFS: Failure-free survival
QOL: Quality of life
GU: Genitourinary
GI: Gastrointestinal
EBRT: External beam radiation therapy
IMRT: Intensity-modulated radiation therapy
VMAT: Volumetric modulated arc therapy
CTCAE: Common Terminology Criteria for Adverse Events
RTOG: Radiation Therapy Oncology Group
HDR: High-dose rate
LF: Local failure
CT: Computed tomography
MCIC: Minimal clinically important change
MR: Magnetic resonance
MR-LINAC: Magnetic resonance linear accelerator
OAR: Organs-at-risk
MRgRT: Magnetic resonance-guided radiotherapy
EPIC: Expanded Prostate Cancer Index Composite
TTP: Time to progression
BF: Biochemical failure
RF: Regional failure
DM: Distant metastasis
PCSM: Prostate cancer-specific mortality
FFBF: Freedom from biochemical failure
PSA: Prostate-specific antigen
OS: Overall survival
NCCN: National Comprehensive Cancer Network
ECOG: Eastern Cooperative Oncology Group
IPSS: International Prostatism Symptom Score
TURP: Transurethral resection of the prostate
PSMA: Prostate-specific membrane antigen
PET: Positron emission tomography
SIB: Simultaneous integrated boost
PTV: Planning target volume
CTV: Clinical target volume
PRV: Planning organs-at-risk volume
TRUFI: True fast imaging with steady-state free progression
ADT: Androgen deprivation therapy
EQ-5D: EuroQol-5D Index
AE: Adverse event
SAE: Serious adverse event
IRB: Institutional Review Board
REDCap: Research Electronic Data Capture
CTSC: Clinical and Translational Science Center
DSMB: Data and Safety Monitoring Board
ANOVA: Analysis of covariance
HRs: Hazard ratios
BED: Biologically Effective Dose
EQD2: Equivalence dose in 2 Gy Fractions
