# Supplementary material for: A randomized phase II trial of MR-guided prostate stereotactic body radiotherapy administered in 5 or 2 fractions for localized prostate cancer (FORT)
Source: BMC Cancer. 2023 Sep 30;23:923. doi: 10.1186/s12885-023-11430-z (PMC10544147; doi:10.1186/s12885-023-11430-z)
Supplement: Supplementary file 1 — Additional file 1: Supplemental Table 1. MR-guided Prostate Stereotactic Body Radiation Therapy Organ-at-Risk Constraints and Planning Parameters for 2-fraction and 5-fraction Regimens. [file 12885_2023_11430_MOESM1_ESM.pdf]

Supplemental Table 1. MR-guided Prostate Stereotactic Body Radiation Therapy Organ-at-Risk Constraints and Planning Parameters for 2-fraction and 5-fraction Regimens

Structure	Dosimetric Parameter	Per Protocol
	**5 FRACTION ARM**	
**Structure**	**Dosimetric Parameter**	**Per Protocol**
Rectum	V38.06	< 0.03 cc (Optimal)
	V34.4	< 3 cc (Optimal)
	V32.63	< 10% (Mandatory)
	V29	< 20% (Mandatory)
	V18.3	< 50% (Mandatory)
Bladder	V38.06	< 0.03 cc (Optimal)
	V18.12	< 10% (Mandatory)
Bladder Wall	V38	< 0.03 cc (Optimal)
	V18.3	< 15 cc (Optimal)
Urethra	V38.78	< 0.03 (Optimal)
Penile Bulb	V19.9	< 3 cc (Optimal)
Femoral Head (each)	V19.9	< 1 cc (Optimal)
	V15.6	< 10 cc (Optimal)
	**2 FRACTION ARM**	
**Structure**	**Dosimetric Parameter**	**Per Protocol**
Rectum	V13	< 7 cc (Optimal)
	V17.6	< 4 cc (Optimal)
	V20.8	< 1 cc (Mandatory)
	*Max Point Dose	< 27Gy (Optimal)
Bladder	V14.6	< 15 cc (Mandatory)
	V20.8	< 5 cc (Mandatory)
	*Max Point Dose	< 27Gy (Optimal)
Bladder Wall	V14.6	< 15 cc (Optimal)
	V20.8	< 5 cc (Optimal)
	*Max Point Dose	< 27Gy (Optimal)
Urethra	< 27Gy	D10% (Mandatory)
Penile Bulb	V19.5	< 50% (Optimal)
Femoral Heads	V14	< 10 cc (Optimal)
